# A rare case of aortitis presenting as chest pain: a case report and literature review

**DOI:** 10.1097/MS9.0000000000002140

**Published:** 2024-05-15

**Authors:** Hasaan Ahmed, Mahmoud Ismayl, Anirudh Palicherla, Ruth Ann Mathew Kalathil, Shivani Vaza, Amjad Kabach, Andrew M. Goldsweig, Ahmed Aboeata

**Affiliations:** a Department of Medicine, Division of Internal Medicine, Creighton University School of Medicine, Omaha, NE; b Department of Medicine, Division of Cardiovascular Disease, Creighton University School of Medicine, Omaha, NE; c Department of Cardiovascular Medicine, Mayo Clinic, Rochester, MN; d Department of Cardiovascular Medicine, Baystate Medical Center, Springfield, MA, USA

**Keywords:** aortitis, case report, chest pain, computed tomography, imaging, smoking

## Abstract

**Introduction and importance:**

Chest pain is a frequent reason patients seek medical attention. The broad spectrum of potential etiologies makes determining the underlying cause of chest pain complex. Among cardiovascular etiologies, aortitis is a rare but life-threatening possibility that should be considered in the differential diagnosis.

**Case presentation:**

A 53-year-old female with a history of smoking presented with progressively worsening chest and epigastric pain over several weeks. She had seen multiple physicians previously for the same symptoms with unremarkable work-ups. Physical examination was notable for severe tenderness upon palpation of her lower abdomen. The electrocardiogram and troponins were unremarkable. Computed tomography of the abdomen revealed aneurysmal dilatation of the abdominal aorta, soft tissue thickening, and surrounding inflammatory stranding, consistent with aortitis. Infectious and autoimmune work-ups were unremarkable. Intravenous steroids were initiated, and her symptoms improved significantly. Her aortitis was attributed to inflammation secondary to chronic smoking.

**Clinical discussion:**

Aortitis is a rare condition with varied clinical presentations. Etiologies of aortitis include infection and non-infectious inflammation. Diagnosis of aortitis requires a thorough clinical assessment and prompt imaging of the aorta, with computed tomography being the preferred imaging modality.

**Conclusion:**

Evaluation for cardiovascular chest pain must extend beyond an electrocardiogram and troponin level. Imaging should be considered in patients with atypical symptoms. Aortitis is a rare but important diagnosis requiring immediate treatment.

## Introduction

Highlights

- Chest pain is a frequent reason patients seek medical attention.
- Aortitis is a rare condition with varied clinical presentations including chest pain.
- Etiologies of aortitis include infection and non-infectious inflammation.
- Diagnosis of aortitis requires a thorough clinical assessment and prompt imaging of the aorta.

Chest pain is a frequent reason patients seek medical attention, encompassing a broad spectrum of eliciting etiologies such as ischemic, pleuritic, gastroesophageal, musculoskeletal, and inflammatory causes^1^. Inflammatory causes of chest pain are inherently multifaceted, consisting of pericarditis, myocarditis, costochondritis, pleuritis, rheumatoid arthritis, and aortitis^1^. Evaluation of chest pain requires a thorough assessment, prioritizing excluding life-threatening conditions before considering less dangerous causes^1^. Diagnosing the underlying cause of chest pain may be challenging due to the overlap between symptoms of various pathologies^1^.

Sharma *et al.* describe aortitis as inflammatory changes of the aortic wall resulting from infectious or inflammatory causes^2^. Aortitis remains a rare disease, with 1–3 cases per million diagnosed annually^3^. Patients afflicted with aortitis often present clinically with fluctuating and non-specific chest pain^2^. Given its rarity and non-specific presentation, aortitis may be under-recognized. We describe a rare case of aortitis, which has been reported in line with CARE criteria, presenting in a 53-year-old female smoker with recurrent chest pain and review the literature regarding contributing etiologies, diagnostic methods, and management, highlighting the importance of early recognition^4^.

## Case presentation

A 53-year-old obese female with a past medical history significant for chronic tobacco smoking presented as a transfer from an outside hospital with recurrent, severe chest and abdominal pain. She had visited emergency departments at multiple hospitals seven times and was discharged with unremarkable work-ups. She denied oral or genital ulcers, photosensitivity, visual disturbances, hearing loss, headaches, and jaw or limb claudication on admission. Vitals were notable for mild hypertension but were otherwise unremarkable. Physical examination was notable for trace pedal edema but no synovitis. No rashes or abnormal nail fold capillaries were appreciated. Tenderness with voluntary guarding was noted upon palpation of all four abdominal quadrants. Limbs were warm without discoloration, and no abdominal bruits were heard. Labs on admission were significant for an elevated absolute neutrophil count (9 k/ul; reference ≤8 k/ul). Urine analysis was negative for infection.

Other than chronic tobacco use, the patient’s past medical history was unremarkable. She had no prior hospitalizations other than for childbirth. She never underwent any surgical intervention. The patient’s family history was noncontributory. She denied any use of illicit or recreational drugs. She was neither diagnosed with any mental-health issues nor was she ever hospitalized due to any mental-health crises.

**Figure 1:**
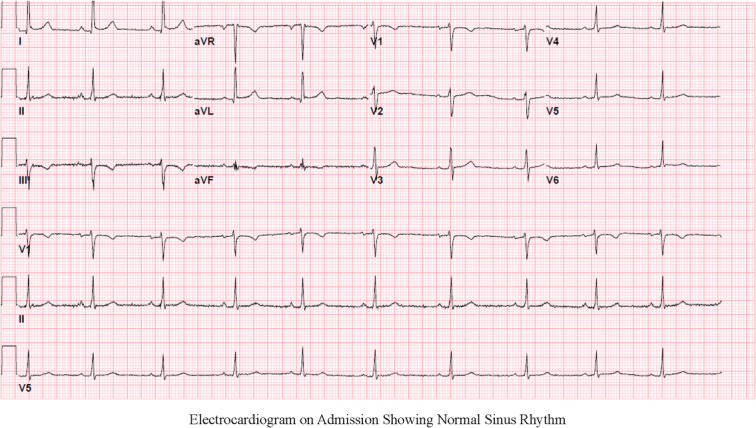
Electrocardiogram on admission showing normal sinus rhythm.

Due to the patient’s chest pain and her risk factors of obesity and chronic smoking, a basic cardiac work-up was conducted due to the initial concern for acute coronary syndrome. An electrocardiogram on admission showed normal sinus rhythm (Fig. 1), and a transthoracic echocardiogram showed normal ejection fraction with no wall motion abnormalities. High-sensitivity troponin (8.4 ng/l; reference ≤52.0 ng/l) and B-type natriuretic peptide (<30 pg/ml; reference ≤124 pg/ml) were both unremarkable.

**Figure 2:**
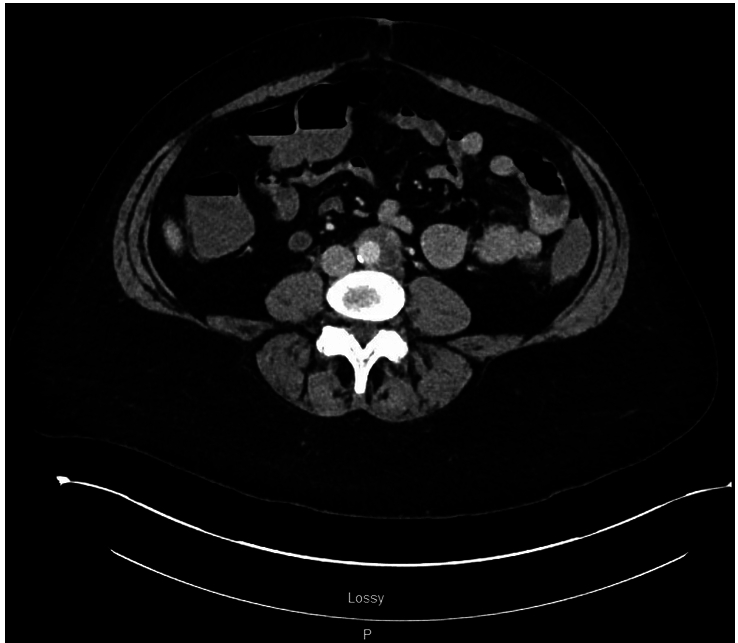
Computed tomography abdomen and pelvis revealing crescentic soft tissue infiltration in the distal abdominal aorta with surrounding inflammatory stranding extending to the left common iliac artery.

**Figure 3:**
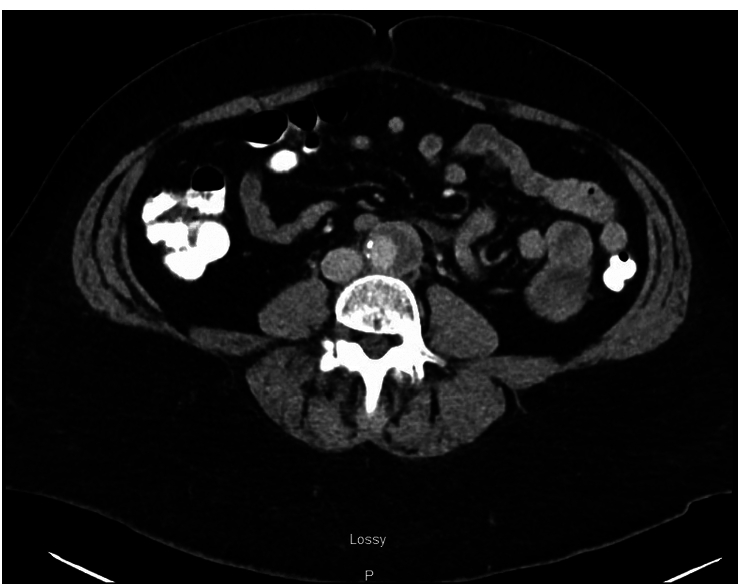
Computed tomography angiogram of the abdomen and pelvis showing circumferential soft tissue thickening in the distal aorta, suggestive of aortitis.

Because her cardiac work-up was suggestive of non-ischemic chest pain, other diagnoses were explored such as infectious, psychological, inflammatory, and somatic etiologies. Computed tomography (CT) of the abdomen and pelvis with contrast was obtained due to the patient’s abdominal pain, revealing crescentic soft tissue infiltration in the distal abdominal aorta with surrounding inflammatory stranding extending to the left common iliac artery (Fig. 2). Subsequent CT angiogram (CTA) of the abdomen and pelvis with contrast revealed circumferential soft tissue thickening in the distal aorta, suggestive of aortitis (Fig. 3). Due to concern for an infectious etiology, two sets of blood cultures were obtained and showed no growth. Vasculitis work-up led by the rheumatology service consisted of C3 complement, C4 complement, qualitative anti-nuclear antibody screen, erythrocyte sedimentation rate, C-reactive protein, rheumatoid factor, and anti-neutrophilic cytoplasmic antibody and was unremarkable. Intravenous methylprednisolone 60 mg daily was initiated to address her inflammation, and as-needed ketorolac was started to manage her pain. Given her negative vasculitis and infectious work-up, her aortitis was determined to be secondary to chronic inflammation from smoking, and she was counseled extensively about smoking cessation. She was discharged on a 4-week course of prednisone 60 mg daily and a seven-day course of as-needed naproxen.

Given that the patient was admitted to a university academic medical center, there were no limitations concerning access to consultants, laboratory diagnostics, imaging, or potential procedural intervention. There were no financial or cultural limitations hindering our ability to diagnose the patient. However, given that the patient was from a rural part of the state, we believe that geographical limitations in accessing resources as well as healthcare professionals may limited her diagnostic work-up.

The patient presented for post-hospitalization follow-up 1 month after discharge, endorsing significant improvement in her chest and abdominal pain in the setting of medical therapy and smoking cessation. She also endorsed minimal use of over-the-counter analgesics. Steroids were tapered, and she was instructed to follow-up as needed. The patient’s perspective was that of immense gratitude knowing that the cause behind her prolonged duration of chest and abdominal pain was finally identified and treated.

## Discussion

Aortitis presents clinically with non-specific and fluctuating symptoms, making diagnosis a challenge^5^. Patients may present with fatigue, malaise, and fever in the setting of chest and/or abdominal pain, with aortic rupture and aortic dissection being the most severe complications^5,6^. Based on the specific area of aortic involvement, other clinical conditions may occur such as stroke, myocardial infarction, mesenteric ischemia, renal failure, and congestive heart failure, further complicating the diagnosis^5^. Given the variability of aortitis symptoms and its risk for progression to life-threatening conditions, a timely and accurate diagnosis is imperative^6^.

Aortitis can be classified into two pathophysiologic groups, infectious and non-infectious, with the majority of aortitis being non-infectious in etiology^5,6^. Infectious aortitis is characterized by inflammation of the aorta, typically incited by a virus, bacteria, or fungus^5,6^. Non-infectious aortitis is characterized by sterile inflammation of the aorta, with rheumatologic disorders such as giant cell arteritis and Takayasu arteritis being the most common causes^5,6^. Following a negative rheumatologic work-up, our patient’s aortitis was attributed to the chronic inflammatory effects of cigarette smoking^7,8^.

Cigarette smoking remains a major risk factor for cardiovascular disease, with the *Centers for Disease Control and Prevention* estimating nearly 12% of the United States population are active smokers^9,10^. The detrimental impact of cigarette smoking on cardiovascular outcomes is multifactorial, with inflammation, oxidative stress, and endothelial dysfunction contributing^9^. Prior studies have investigated the relationship between cigarette smoking and inflammation as seen in a cross-sectional study by McEvo*y et al.* ^11^, who found current cigarette smokers had significantly higher inflammatory markers such as high-sensitivity C-reactive protein, interleukin-6, and fibrinogen (*P*<0.001) compared to non-smokers. Furthermore, McEvo*y et al.* ^11^ noted that inflammatory markers incrementally increased among current smokers when stratified by pack-year history, highlighting the cumulative impact of tobacco exposure. Smoking specifically confers a higher risk of adverse cardiovascular outcomes among middle-aged women, driven by inflammation, as highlighted in a cross-sectional analysis by Bermudez *et al.* ^12^, who noted increasing levels of plasma inflammatory markers of vascular inflammation such as E-selectin (*P*=0.036), hs-CRP (*P*=0.034), IL-6 (*P*=0.002), and sICAM-1 (*P*<0.001) among female middle-aged smokers compared to non-smokers. Vascular inflammation is further amplified by reactive oxygen species formed during smoking, disrupting homeostatic vascular signaling while promoting pro-inflammatory processes in the endothelium^9^. In essence, systemic inflammatory processes are elevated in women who are active cigarette smokers compared to non-smokers, suggesting that smoking is likely an independent promotor of vascular inflammation and aortitis^12^.

Given that aortitis mimics many major cardiovascular diseases, a thorough diagnostic evaluation is warranted when aortitis is clinically suspected^5,6^. Non-invasive imaging techniques are essential for diagnosing aortitis, including ultrasound, CTA, magnetic resonance angiography (MRA), and positron emission tomography (PET)^5,6^. In the case of our patient, CTA with iodinated contrast was used to assess for aortitis, demonstrating the thickening of the aortic wall, while excluding life-threatening conditions such as aortic dissection and intramural hematoma^6^. The role of CT extends beyond the initial diagnostics of aortitis, as CT is often used for assessing the resolution of aortitis following treatment initiation^6^. Laboratory testing in aortitis is important to identify the potential etiology^6^. Initial lab work in patients who have a high suspicion of aortitis should consist of inflammatory markers such as C-reactive protein, erythrocyte sedimentation rate, a complete metabolic panel, and blood cultures to exclude infectious causes^6^. Rheumatological testing such as anti-neutrophil cytoplasmic antibodies, rheumatoid factor, and anti-nuclear antibodies may also be beneficial^13^. While our patient’s laboratory work-up was unremarkable, this did not preclude her from being diagnosed with aortitis, as aortitis is diagnosed based on a combination of clinical symptoms and imaging findings^6^.

Management of aortitis is centered around halting disease progression while decreasing the likelihood of complications, with treatment varying based on the underlying cause^3^. In patients with infectious aortitis, treatment consists of broad-spectrum antibiotics, which should eventually be narrowed to treat the suspected organism, with the optimal duration of treatment varying based on the patient’s immunological status^3^. In rare instances of fulminant infectious aortitis complicated by aortic rupture, aortic surgery may be necessary^3^. In patients with non-infectious aortitis, immunosuppressive therapy remains the cornerstone of management in suppressing the inflammation^6^. High-dose glucocorticoid therapy is typically initiated followed by a reduced dose with symptom control^3^. In patients whose symptoms remain poorly controlled or who become intolerant to glucocorticoids, adjunctive therapies with interleukin-6 receptor antagonists or disease-modifying antirheumatic drugs have been used^3^. Patients with non-infectious aortitis are often managed by a multidisciplinary healthcare team consisting of a cardiologist, a rheumatologist, and a surgical specialist and are followed over months to years to assess their response to pharmacotherapy^6^.

Our case report has several strengths and limitations. One strength is that our case report serves as an educational platform to highlight the rarity of aortitis and the need for providers to think beyond cardiac work-up in patients who present with chest. Additionally, our case report serves as an aid for clinical guidance for providers evaluating patients with chest pain of unclear etiology. The limitation of our case report is the verifiability of whether other causes were also contributing to the patient’s symptoms, in the context of her multiple emergency room visits, such as psychological or musculoskeletal disorders. The fact that our patient never underwent any stress testing or imaging with multispiral CT coronary angiography, for further evaluation of her chest pain, prevents us from definitively excluding coronary artery disease as a potential contributor to her symptoms. Another limitation is that being that this is a case report, there is no control to evaluate the effects of smoking cessation on chest pain. Furthermore, there is a limited number of comparative studies that have evaluated smoking cessation in aortitis patients specifically, making our case report primarily hypothesis-generating. Future comparative studies are needed to assess the impact of smoking among patients with aortitis.

## Conclusion

Aortitis is a rare clinical condition with infectious and inflammatory causes. The present case emphasizes the importance of evaluating chest pain beyond electrocardiograms and troponins, with the need to consider imaging to establish a definitive diagnosis. Given the non-specific symptoms of aortitis, which often overlap with other conditions, physicians should have a low threshold to screen for aortitis in patients presenting with chest pain.

## Ethical approval

Ethics clearance was not necessary as this was a case report. There was no research study conducted and Institutional Review Board approval was not needed.

## Consent

Written informed consent was obtained from the patient for publication of this case report and accompanying images. A copy of the written consent is available for review by the Editor-in-Chief of this journal on request.

## Source of funding

No funding was sought or utilized for this manuscript.

## Author contribution

Conceptualization: H.A., M.I., A.P., A.M.G., A.K., A.A.; writing—original draft: H.A., M.I., A.P., A.M.G.; writing—review and editing: A.M.G., A.K., A.A.; investigation: H.A., M.I., A.P.; supervision: A.M.G., A.K., A.A.

## Conflicts of interest disclosure

Dr Goldsweig reports speaking for Philips and Edwards Lifesciences and consulting for Philips and Inari Medical.

## Research registration unique identifying number (UIN)

Not applicable.

## Guarantor

Hasaan Ahmed.

## Data availability statement

We, the authors, have nothing to declare in this category as it is not applicable.

## Provenance and peer review

Not commissioned, externally peer-reviewed.
